# The Association Between Toddlers’ Temperament and Well-Being in Norwegian Early Childhood Education and Care, and the Moderating Effect of Center-Based Daycare Process Quality

**DOI:** 10.3389/fpsyg.2021.763682

**Published:** 2021-12-06

**Authors:** Catharina P. J. van Trijp, Ratib Lekhal, May Britt Drugli, Veslemøy Rydland, Suzanne van Gils, Harriet J. Vermeer, Elisabet Solheim Buøen

**Affiliations:** ^1^Department of Communication and Culture, BI Norwegian Business School, Oslo, Norway; ^2^Department of Education, University of Oslo, Oslo, Norway; ^3^Regional Center for Child and Adolescent Mental Health – Central Norway, Norwegian University of Science and Technology, Trondheim, Norway; ^4^Center for the Study of Educational Practice, Inland Norway University of Applied Sciences, Elverum, Norway; ^5^Center for Education and Child Studies, Leiden University, Leiden, Netherlands; ^6^Center for Child and Adolescent Mental Health, Eastern and Southern Norway, Oslo, Norway

**Keywords:** temperament, well-being, process quality, ECEC, Norway, toddlers

## Abstract

Children who experience well-being are engaging more confidently and positively with their caregiver(s) and peers, which helps them to profit more from available learning opportunities and support current and later life outcomes. The goodness-of-fit theory suggests that children’s well-being might be a result of the interplay between their temperament and the environment. However, there is a lack of studies that examined the association between children’s temperament and well-being in early childhood education and care (ECEC), and whether this association is affected by ECEC process quality. Using a multilevel random coefficient approach, this study examines the association between toddlers’ (*N* = 1,561) temperament (shyness, emotionality, sociability, and activity) and well-being in Norwegian ECEC and investigates whether process quality moderates this association. Results reveal an association between temperament and well-being. Staff-child conflict moderates the association between shyness and well-being, and between activity and well-being. Moreover, high emotional behavioral support moderates the association between activity and well-being. Extra attention should be paid by the staff to these children’s needs.

## Introduction

Children’s social-emotional well-being (“well-being”) promotes children’s current and later developmental and learning outcomes (Mashford-Scott et al., 2012). Well-being is to a large extent determined by children’s experiences in their environment. The neuroplasticity of the brain causes children to be highly sensitive to the level of support from their environment, especially during their first years of life (Blakemore and Frith, 2005; National Scientific Council on the Developing Child, 2007). As a result, a strong feeling of well-being supports children to engage more confidently and positively with their environment, which helps them to profit more from available learning opportunities (Department for Education and Child Development, 2016; La Paro and Gloeckler, 2016). Children spend considerable time in early childhood education and care (ECEC) settings (Council of the European Union, 2019). In Norway, 85.4% of the children aged 1–2 years attended an ECEC center in 2020. Most of these children (96.3%) spend 41 h or more per week in the ECEC center (Statistics Norway, 2021). This high fulltime attendance rate underlines the importance of studying children’s well-being and its predictors in ECEC. Well-being is a key concept in many (inter)national ECEC quality frameworks and guidelines (Mashford-Scott et al., 2012; Council of the European Union, 2019). ECEC plays a key role in children’s lives by building a foundation for health, well-being, higher level competence development, and educational success (Council of the European Union, 2019). Previous studies have shown that high-process-quality ECEC (e.g., high-quality staff-child interactions) supports children’s current and later well-being directly (e.g., Helmerhorst et al., 2014; Melhuish et al., 2015).

Other studies, building on theoretical frameworks, suggest that children’s outcomes might be a result of the interplay between children’s characteristics and their environment. For instance, Thomas and Chess (1977) argued that goodness-of-fit is the compatibility between a child’s temperament and the environment, whereas poorness-of-fit occurs when there is a discrepancy between a child’s temperament and the environmental expectations and opportunities. Rothbart and Bates (1998) defined children’s temperament as constitutional individual differences in self-regulation, attentional, emotional, and motor reactivity. Considering the goodness-of-fit theory and the importance of well-being for children’s current and later life outcomes, more studies are needed to gain knowledge about how children’s well-being is affected by both children’s temperament and ECEC process quality. The present study therefore examines the association between children’s temperament and well-being in Norwegian ECEC, and the possible moderating effect of ECEC process quality. The findings will provide insights on which children with certain temperamental styles might need extra attention if they are to experience a high level of well-being, and on how ECEC process quality can promote these higher levels of well-being.

Most studies that examined the interplay between children’s temperament and the environment in developmental outcomes have focused on school-aged children (e.g., Holder and Klassen, 2010) or on outcomes such as social-emotional development (e.g., Hipson and Séguin, 2016). Holder and Klassen (2010) found that, depending on the measures, temperament accounted for 9–29% of the variance in happiness in children aged 9–12 years. Children who were less shy, anxious, and emotional, and more active and social were happier (Holder and Klassen, 2010). Both happiness and temperament are partially heritable and relatively stable but also follow a developmental process through experience, which might cause some changes in the level of temperament and happiness (Buss and Plomin, 1984; Shiner, 1998; Røysamb et al., 2014; Nes and Røysamb, 2017). However, studies on the youngest children and the association between their temperament and well-being in ECEC are underrepresented. To our knowledge, only two studies by De Schipper et al. (2003, 2004) examined the role of children’s temperament in relation to children’s well-being in ECEC. Both studies used the Leiden Inventory for the Child’s Well-Being in Day Care (LICW-D) to measure four aspects of well-being in day care: general well-being, well-being with professional caregivers, well-being in the physical setting of the ECEC center, and well-being with peers. The inventory was based on the Well-being Scale used in an earlier study by Van IJzendoorn et al. (1998). De Schipper et al. (2003) found that children (aged 6–30 months) with a more difficult temperament (e.g., more difficulty to adapt to novelty and showing more irritable distress) showed a lower level of well-being. In addition, based on the same sample, De Schipper et al. (2004) found that children with an easier temperament showed more well-being.

Studies that focused on the direct effect of ECEC process quality on young children’s well-being found that staff-child interactions, relations with peers (Bjørgen, 2015; Sandseter and Seland, 2017), and environmental chaos (Werner et al., 2015) had an effect on well-being. Groeneveld et al. (2010) found that caregiver sensitivity had a positive effect on children’s well-being in home-based childcare but not in center care; this could be explained by the fact that children in center care have more than one caregiver, and the individual differences of caregivers were not taken into account. Nevertheless, De Schipper et al. (2004) found that children with a more easy-going temperament showed more well-being and also that greater availability of trusted professional caregivers affected the association between children’s temperament and well-being in ECEC, as this helped children adapt more easily to the care setting. Thus, considering Thomas and Chess’ (1977) goodness-of-fit-theory and the studies mentioned above, it might be that the potential association between children’s temperament and well-being in ECEC is affected by ECEC process quality. However, as argued by Chess and Thomas (1984), to be able to reach goodness-of-fit, the caregivers should create an environment for the child that matches the child’s temperament. Children might attempt to change the environment to suit their own temperament, which is a behavioral strategy and attempt to cope with a stressful conflict that they cannot master directly. Therefore, caregivers should be responsive to children’s needs and modify the environment if needed (Chess and Thomas, 1984).

This study investigates the association between children’s temperament and well-being in ECEC in a large sample of young children aged 1–3 years in ECEC centers (center-based daycare) in Norway. Using a multilevel random coefficient modeling approach, we examine the following research questions: (1) Is there an association between children’s temperament and well-being in Norwegian ECEC?, and (2) Does ECEC process quality moderate the association between children’s temperament and well-being in Norwegian ECEC? We expect to find that children who are less shy and emotional and more social and active experience more well-being. In addition, we expect that process quality—namely, staff-child relationship, emotional and behavioral support, and chaos in the group—moderates the association between temperament and well-being.

## Materials and Methods

### Recruitment and Participants

The data for the present study were derived from the first round of data collection (baseline data) from the larger Thrive by 3 study (Trygg før 3) (Lekhal et al., 2020). Thrive by 3 is a cluster randomized controlled trial studying a multicomponent professional development intervention that was developed to enhance process quality in toddler classrooms in Norwegian ECEC centers. As a result of enhanced process quality, the goal is to strengthen the mental health, development, and well-being of children aged 1–3 years. Four municipalities/city districts in Eastern Norway and three in Central Norway were invited and consented to participate in the study. The mangers of the ECEC centers, professional caregivers, parents, and children were invited by e-mail (or letter, if needed) with an electronic link to the written consent form. The managers of the ECEC decided on the ECEC center’s participation and their own participation. A total of 78 ECEC centers and 187 units/groups agreed to participate. In addition, the managers forwarded, on behalf of the Thrive by 3 study, the written consent form to all professional caregivers, parents, and children at the center. Parents consented on behalf of their children; both parents needed to consent. A written consent was provided for 1,561 children (800 boys, 761 girls), aged 7 to 43 months (*M* = 21.4 months, *SD* = 6.2), who were part of 185 units/groups. The study was approved by the Regional Committees for Medical and Health Research Ethics South East Norway and by the Norwegian Centre for Research Data.

The first data collection round took place at the beginning of the childcare year (September 2018), and some of the children had just started in ECEC. The professional caregiver who knew the child best filled out an electronic questionnaire on the child’s well-being and the staff-child relationship. One of the parents (1,114 mothers and 447 fathers) filled out the electronic questionnaire on child and family characteristics and the child’s temperament. The process quality in the classroom was measured using both questionnaire data from the staff in the unit/group and observations by external observers who also work in ECEC.

### Non-response

As we collected data from multiple respondents using differing questionnaires, we had missing at random (MAR) (Rubin, 1976). The missing patterns were tested with IBM SPSS Statistics Version 27.0 (IBM Corporation, 2020) and showed that 1,264 children (81% of 1,561 children) had complete data on all variables. The most common missing patterns were caused by missings on the temperament scales or child and family variables. This was often because the parent did not fill out the questionnaire for the child. Table 1 shows the descriptives, including the number of missings for each variable. Data was not imputed; instead, maximum likelihood with robust standard errors (MLR) was used as an estimation method to cope with missings.

**TABLE 1:** Descriptives: child and family characteristics, temperament, ECEC process quality, and child well-being variables.

	**%/*M***	***SD***	***n***	***n* missing**	**Cronbach’s alpha**
**Level 1**					
Gender			1,561	0	
Boys	51.2%		800		
Girls	48.8%		761		
Age in months	21.4	6.2	1,558	3	
Language			1,365	196	
Norwegian	91.4%		1,247		
Minority language	8.6%		118		
Hours per day in ECEC^1^	2.12	0.41	1,354	207	
Family gross income^2^	5.00	1.22	1,358	203	
Staff-child relationship, closeness scale	4.35	0.50	1,471	90	0.71
Staff-child relationship, conflict scale	1.50	0.54	1,471	90	0.74
Well-being	4.45	0.45	1,472	89	0.82
Shyness	2.45	0.64	1,321	240	0.74
Emotionality	2.73	0.67	1,321	240	0.79
Sociability	3.64	0.53	1,321	240	0.58
Activity	3.93	0.57	1,322	239	0.71
**Level 2**					
Emotional and behavioral support	5.84	0.71	185	0	0.88
Environmental chaos in the group	2.07	0.38	185	0	0.87

*Internal consistency based on Cronbach’s alpha (α): α < 0.50 unacceptable; 0.50 ≤ α < 0.60 poor; 0.60 ≤ α < 0.70 acceptable; 0.70 ≤ α < 0.90 good; α ≥ 0.90 excellent.*

*^1^Answer categories 1 = less than 6 h, 2 = 6–8 h, and 3 = more than 8 h.*

*^2^Answer categories 1 = under 200,000, 2 = 200,000–399,000, 3 = 400,000–599,000, 4 = 600,000–799,000, 5 = 800,000–999,000, and 6 = over 1,000,000 Norwegian kroner.*

### Measures

#### Temperament

##### Emotionality, Activity, Sociability (EAS) temperament survey for children

Children’s temperament was studied using the EAS Temperament Survey for Children (EAS; Buss and Plomin, 1984) filled out by the parent. The questionnaire consisted of four subscales: shyness (e.g., child becomes shy easily; trusts strangers very easily), emotionality (e.g., child cries easily; gets flustered easily), sociability (e.g., child likes being with other people; does not like being alone), and activity (e.g., child is always on the go; is full of energy). Each subscale was assessed by 5 items answered on a 5-point Likert scale ranging from 1 (*very typical*) to 5 (*not at all typical*). A high score on shyness, emotionality, sociability, and activity meant that the child was more shy, emotional, social, and active. For the subsequent analyses, the individual mean scores on each scale of the EAS were group mean centered (score of the child compared to the other children in their unit/group) for the within level and aggregated (mean score for the whole unit/group) for the between level. The latter was used to control for the between level.

#### Well-Being in Early Childhood Education and Care

##### Leiden inventory for the child’s well-being in daycare

The LICW-D (De Schipper et al., 2004) filled out by the professional caregiver who knew the child best was used to assess children’s well-being in ECEC. The 12 items were answered on a 5-point Likert scale ranging from 1 (*never*) to 5 (*always*), and the questionnaire had been validated in an earlier Norwegian study (Van Trijp et al., 2021). The LICW-D items assess children’s general well-being (e.g., child enjoys attending the daycare center), and how comfortable the child is with the professional caregiver(s) (e.g., child is happy to see the professional caregiver(s) when he/she is dropped off), peers (e.g., child trusts all the children at the daycare center), and the physical setting of the center (e.g., child really enjoys the games and play material at the daycare center). A higher score meant a higher level of well-being. For subsequent analyses, the individual mean scores were used for each child on the whole scale of the LICW-D.

#### Early Childhood Education and Care Process Quality

##### Student teacher relationship scale – short form

The Student Teacher Relationship Scale – Short Form (STRS-SF; Pianta, 2001), a teacher report instrument, was filled out by the professional caregiver who knew the child best and assessed the relationship. The questionnaire consisted of two subscales: closeness (8 items, e.g., I share an affectionate, warm relationship with this child; when I praise this child, he/she beams with pride), and conflict (7 items, e.g., this child and I always seem to be struggling with each other; the child easily becomes angry at me). Items were answered on a 5-point Likert scale ranging from 1 (*definitely does not apply*) to 5 (*definitely applies*). A high score on closeness meant that the professional caregiver had a close relationship with the child; a high score on conflict meant that there was a conflictual relationship between the professional caregiver and child. For subsequent analyses, the individual mean scores for both the closeness and conflict scale were group mean centered for the within level and aggregated for the between level.

##### Classroom assessment scoring system

The quality of the staff-child interactions in the groups was assessed by an observation instrument, the Classroom Assessment Scoring System (CLASS), Toddler version (La Paro et al., 2012). The observations were made by 24 trained and certified CLASS Toddler observers. Each observation consisted of three rounds of 15 min, starting at 8:30 a.m. All three rounds were scored separately by the same observer. CLASS Toddler consists of two domains: (1) emotional and behavioral support, and (2) engaged support for learning. For the present study, we focused only on the emotional and behavioral support domain, as earlier studies showed that well-being is affected by relations with others and the atmosphere in the group rather than by learning dimensions (Groeneveld et al., 2010; Bjørgen, 2015; Werner et al., 2015; Sandseter and Seland, 2017). This domain consisted of five dimensions: positive climate, negative climate (reversed), teacher sensitivity, regard for child perspectives, and behavior guidance. All dimensions were rated on a 7-point scale ranging from low to high. A high score meant a higher level of emotional and behavioral support in the group, and thus better staff-child interactions on the group level. Eighteen units/groups (10.3% of the total observations) were observed by two observers. The interrater reliability for observations with two observers was 88.3% for the emotional and behavioral support domain. The mean scores of the five dimensions were computed to a total mean score for the emotional and behavioral support domain. The total mean score was grand mean centered (score of the child compared to the whole sample) for the moderation analyses.

##### Life in early childhood programs

The Life in Early Childhood Programs (LECP; Kontos and Wachs, 2000), filled out by the professional caregivers in the child’s unit/group, was used to examine the environmental chaos in the group. The level of chaos in the group was calculated by the mean score of all professional caregivers answered that for a certain group. The 16 items were answered on a 5-point Likert scale ranging from 1 (*not true*) to 5 (*very often true*) and asked for professional caregivers’ views on the degree of organization and control in the group (e.g., degree of consistency or routines; whether things are placed in the same place), use of space, group density, and environmental traffic (whether many people come and go). A higher score meant a higher level of chaos in the group. We computed the mean scores of the 16 items to a mean score for LECP. This mean score was grand mean centered for the moderation analyses.

#### Child Characteristics

For the first research question, we controlled for the following child characteristics that were filled out by the parent: Child’s gender (0 = boys, 1 = girls), child’s age in months, whether the child has a Norwegian or other linguistic background (answer categories 1 = Norwegian, 2 = minority language from a Western country in Europe or North America, 3 = minority language from a non-Western country), and the number of hours in the ECEC center per day (answer categories 1 = less than 6 h, 2 = 6 h, 3 = 6–8 h, and 4 = more than 8 h). For subsequent analyses, the answer categories of linguistic background (0 = Norwegian, 1 = minority language) and the number of hours in the ECEC center (1 = less than 6, 2 = 6–8 h, 3 = more than 8 h) were computed. In addition to the child’s language, the child’s gender was treated as a dummy variable. The child’s age in months and number of hours in the ECEC center per day were grand mean centered, because we wanted to compare the child’s score to the whole sample.

Note that we also collected data on if the child has any kind of disability (0 = no, 1 = yes), which was filled out by the parent. However, out of the 1,365 parent answers, only 1.5% of the children were answered with ‘yes’. We conducted initial analyses to examine if we should include the child’s disability as a control variable for research question one, but the results showed that there was no effect of this variable. Due to these findings and the low prevalence of children with a disability, we decided not to include the ‘child’s disability’ as a control variable.

#### Family Characteristics

In addition to the child’s characteristics, we controlled for one family characteristic that was filled out by the parent, namely: What the family’s total gross year income is (answer categories 1 = under 200,000, 2 = 200,000–399,000, 3 = 400,000–599,000, 4 = 600,000–799,000, 5 = 800,000–999,000, and 6 = over 1,000,000 Norwegian kroner). This variable was grand mean centered for subsequent analyses.

### Analyses

The research questions were tested using multilevel random coefficient modeling with the MLR estimator in Mplus Version 8 (Muthén and Muthén, 2017). For our first research question, we were interested in the within level effect of children’s temperament on well-being. Both the outcome variable children’s well-being and the predictors shyness, emotionality, sociability, and activity were level 1 variables (child level). To test the strength of this association, we wanted to add control variables. Following recent recommendations on inclusion of meaningful control variables (Bernerth and Aguinis, 2016; Sturman et al., 2021), we examined the correlations between child and family characteristics, the different temperament scales and well-being to identify demographic variables that showed significant correlations with either the predictors and/or outcome variable (see Table 2). Those variables were included in a subsequent model as control variables. This resulted in two models: Model 1 was uncontrolled, and model 2 was controlled for all child and family characteristics that are presented in Table 2. In addition, following recommendations by Preacher et al. (2016) we separated within- and between-level effects by person-mean centering all variables at the child level and including group means at the between level.

**TABLE 2:** Correlations between child and family characteristics, temperament, ECEC process quality, and child well-being variables.

	**Variables**	**1**	**2**	**3**	**4**	**5**	**6**	**7**	**8**	**9**	**10**	**11**	**12**	**13**	**14**
	**Level 1**														
1	Gender	–													
2	Age in months	0.01	–												
3	Language	–0.03	< −0.02	–											
4	Hours per day in ECEC	0.01	0.11***	–0.04	–										
5	Family’s gross income	–0.02	–0.05	−0.24***	0.13***	–									
6	Staff-child relationship, closeness scale	0.04	−0.22***	−0.10***	0.08**	0.08**	–	−0.22***	0.37***	–0.03	0.01	–0.01	−0.06*		
7	Staff-child relationship, conflict scale	–0.02	0.14***	0.02	0.03	–0.02	−0.21***	–	−0.21***	–0.04	0.02	0.01	0.05		
8	Well-being	–0.02	0.11***	−0.07*	0.12***	0.07**	0.43***	−0.24***	–	−0.18***	−0.10**	0.08**	0.05		
9	Shyness	0.03	0.08**	0.08**	–0.02	–0.05	−0.07*	–0.03	−0.20***	–	0.27***	−0.41***	−0.28***		
10	Emotionality	0.05	0.03	< −0.01	0.04	−0.06*	0.01	0.04	−0.09***	0.29***	–	–0.02	0.03		
11	Sociability	0.09**	−0.15***	–0.05	–0.03	0.07*	< −0.01	0.01	0.10***	−0.43***	–0.03	–	0.28***		
12	Activity	−0.07**	−0.08**	−0.05*	0.05	< −0.01	–0.04	0.05	0.05	−0.27***	0.02	0.30***	–		
	**Level 2**														
13	Emotional and behavioral support	0.05	–0.01	–0.04	0.01	–0.02	0.01	–0.03	–0.03	0.04	0.01	–0.05	–0.02	–	
14	Environmental chaos in the group	–0.02	0.08**	–0.04	<0.03	−0.06*	−0.06*	0.14***	−0.15***	0.03	–0.01	–0.04	–0.01	−0.17***	–

*p < 0.05, **p < 0.01, ***p < 0.001 (two-tailed). Correlations at the within level are presented below the diagonal, and correlations at the between level above the diagonal.

Moreover, the model fit was tested to see if the model improved after controlling for more variables. Good model fit was defined as CFI > 0.95, TLI > 0.95, RMSEA ≤ 0.05, and SRMR ≤ 0.05, and acceptable model fit was defined as CFI and TLI.90 –0.95, RMSEA.06 –0.10, and SRMR.06 –0.08 (e.g., MacCallum et al., 1996; Hu and Bentler, 1999). The Akaike information criterion (AIC) and Bayesian information criterion (BIC) were also examined regarding the model fit. If a new model had lower values for AIC and BIC compared to another model, then the model fit was better (Finch and Bolin, 2017). The predictors were analyzed with their group mean centered values, whereas the control variables were either treated as a dummy variable (child’s gender and linguistic background), or grand mean centered (age in months, hours in ECEC, family’s total gross year income). To control for the between level, the predictors were aggregated.

For the second research question, we included both level 1 (child level) and level 2 (unit/group level) ECEC process quality to study their potential moderating effect on the relation between children’s temperament and well-being. All ECEC process quality measures were analyzed separately in a model with one moderator. The predictors were group mean centered, the potential level 1 moderators were group mean centered, and level 2 moderators were grand mean centered. To control for the between group effect, the predictors and level 1 moderators were aggregated. Finally, for the significant interactions, simple slope analyses were conducted to describe the associations between the different scales of temperament and well-being by using ± 1 *SD* of both the predictor and moderator.

## Results

### Preliminary Analyses

Before turning to our research questions, we examined the within- and between group variance components for children’s well-being. The results showed an intraclass correlation coefficient (ICC) of 0.074. This means that 7.4% of the variance in children was due to variance between groups. As this exceeded the suggested 5% threshold (Raudenbush and Liu, 2000; Hox et al., 2018), multilevel analyses were used. The difference between groups is also shown in the significant effects of the intercepts in Tables 3, 4.

**TABLE 3:** Multilevel random coefficient modeling results and fit indices for children’s temperament on well-being in ECEC.

	**Well-being**					
	**Model 1 (*n* = 1,269)^1^**			**Model 2 (*n* = 1,267)^1^**		
	**Estimate (*SE*)**	**AIC**	**BIC**	**Estimate (*SE*)**	**AIC**	**BIC**
Shyness	−0.14*** (0.02)	1,523.76	1,549.48	−0.14*** (0.02)	1,493.60	1,545.04
Intercept	4.83*** (0.15)			4.86*** (0.15)		
Emotionality	−0.07*** (0.02)	1,565.39	1,591.12	−0.07*** (0.02)	1,535.08	1,586.53
Intercept	4.55*** (0.18)			4.60*** (0.18)		
Sociability	0.08** (0.03)	1,562.93	1,588.66	0.09*** (0.03)	1,528.59	1,580.04
Intercept	3.90*** (0.25)			3.84*** (0.25)		
Activity	0.04 (0.02)	1,574.20	1,599.93	0.04 (0.03)	1,546.01	1,597.46
Intercept	4.32*** (0.28)			4.33*** (0.29)		

^1^Children were part of 184 units/groups, and the samples of activity consisted of one more child due to less missings on the activity scale.

*SE, standard error; AIC, Akaike information criterion; BIC, Bayesian information criterion.*

**p < 0.01, ***p < 0.001 (two-tailed).

Model 1 was uncontrolled. Model 2 was controlled for children’s and family characteristics. The intercept for model 0 (intercept-only) was 4.45 (0.02)*** for well-being (*n* = 1,472 children out of 184 units/groups), and AIC = 1,818.66; BIC = 1,834.54. In addition, all models were controlled for the between level.

**TABLE 4:** Moderator models and fit indices: children’s temperament and ECEC process quality on well-being in ECEC.

**Well-being**							
**Effect**	**Estimate**	***SE***	**95% CI**		***p***	**AIC**	**BIC**
			**LL**	**UL**			
Shyness (*n* = 1,267)^1^	−0.153	0.022	−0.197	−0.110	<0.001		
Conflict	−0.228	0.032	−0.290	−0.166	<0.001		
Shyness × Conflict	−0.167	0.050	−0.264	−0.069	0.001	1,427.49	1,468.65
Intercept	5.111	0.179	4.760	5.463	<0.001		
Activity (*n* = 1,268)^1^	0.048	0.23	0.002	0.094	0.041		
Conflict	−0.213	0.032	−0.275	−0.151	<0.001		
Activity × Conflict	0.116	0.056	0.007	0.225	0.037	1,493.38	1,534.55
Intercept	4.561	0.270	4.032	5.091	<0.001		
Activity (*n* = 1,270)^1^	0.039	0.024	−0.007	0.086	0.097		
Emotional behavioral support (EBS)	−0.018	0.021	−0.060	0.024	0.392		
Activity × EBS	0.103	0.032	0.041	0.166	0.001	1,567.25	1,603.28
Intercept	4.323	0.278	3.778	4.868	<0.001		

*^1^Children were part of 184 units/groups.*

*SE, standard error; LL, lower limit; UL, upper limit; CI, confidence interval; AIC, Akaike information criterion; BIC, Bayesian information criterion.*

Table 2 shows the correlations between all variables on both the within and between level. All potential level 1 demographic control variables correlated significantly with a predictor and/or outcome variable at least once. Therefore, all control variables were used in analyzing the relationship between children’s temperament and well-being in ECEC.

### Temperament and Well-Being

Table 3 shows the results for our first research question concerning the association between children’s temperament and well-being in ECEC. A significant negative relationship was found between children’s shyness and well-being and between emotionality and well-being. Children who were more shy or emotional showed a lower level of well-being in ECEC. There was a significant positive relationship between children’s sociability and well-being. Children who were more social showed a higher level of well-being in ECEC. There was no significant relationship between children’s activity and well-being.

As a final step, an inspection of the model fit revealed a good model fit for all models (CFI = 1.00, TLI = 1.00, RMSEA = 0, SRMR = 0). Table 3 shows the model fit regarding the AIC and BIC, which became better when the model included a predictor and was controlled for multiple variables.

### The Role of Early Childhood Education and Care Process Quality

For the second research question, we examined the moderation effects of different level 1 and 2 ECEC process quality on the association between children’s temperament and well-being in ECEC. We found three significant moderation effects, which Table 4 shows.

**FIGURE 1:**
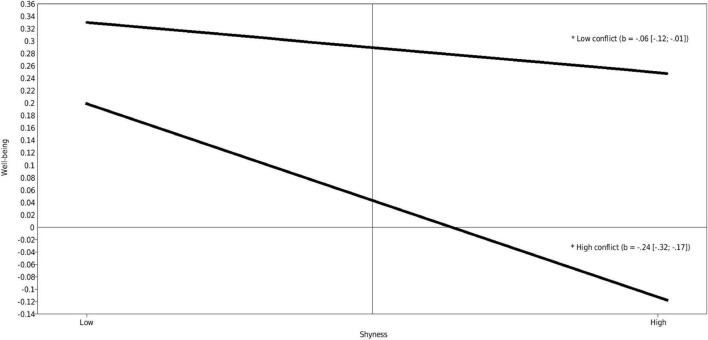
Interactions between shyness and conflict predicting well-being (standardized). Low and high represent –1 SD and +1 SD for both the predictor and moderator.

Conflict in the relationship between the professional caregiver and child had a significant negative moderating effect on the association between children’s shyness and well-being (estimate −0.17, *p* = 0.001). Simple slope analyses revealed a significant negative effect, whereby children who were shyer showed less well-being when they experienced a low (estimate = −0.06, *p* = 0.026) or high (estimate = −0.24, *p* = ≤ 0.001) conflictual relationship with the professional caregiver. Figure 1 provides an illustration. Subsequent analyses showed that the difference between low and high conflict was significant for both children scoring low on shyness (estimate = −0.12, *p* = 0.001) and children scoring high on shyness (estimate = −0.34, *p* = ≤ 0.001), whereby the difference was larger for highly shy children.

**FIGURE 2:**
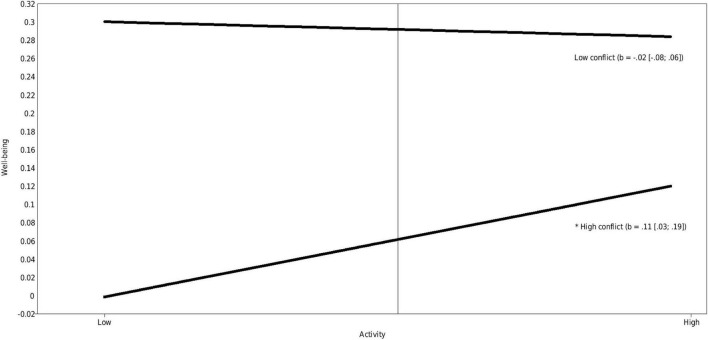
Interactions between activity and conflict predicting well-being (standardized). Low and high represent –1 SD and +1 SD for both the predictor and moderator.

Conflict also had a significant positive moderating effect on the association between children’s activity and well-being (estimate = 0.12, *p* = 0.037). Simple slope analyses revealed a significant positive effect of activity and well-being for high conflict (estimate = 0.11, *p* = 0.006). This means that there was an association between children’s activity and well-being only when the level of conflict in their relationship with the professional caregiver was high, whereby more active children showed more well-being. Figure 2 presents an illustration. Subsequent analyses showed that the difference between low and high conflict was significant for both low active (estimate = −0.28, *p* = ≤ 0.001) and highly active children (estimate = −0.15, *p* = ≤ 0.001), whereby the difference was larger for low active children.

**FIGURE 3:**
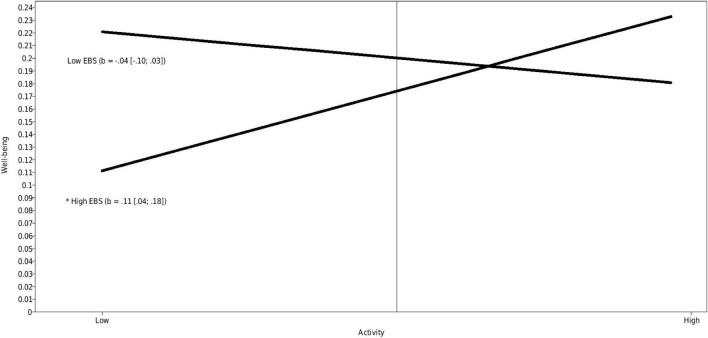
Interactions between activity and emotional and behavioral support predicting well-being (standardized). Low and high represent –1 SD and +1 SD for both the predictor and moderator.

Emotional and behavioral support had a significant positive moderating effect on the association between children’s activity and well-being (estimate = 0.10, *p* = 0.001). Simple slope analyses revealed a significant positive effect for high emotional and behavioral support (estimate = 0.11, *p* = 0.001). This means that there was an association between children’s activity and well-being only when the level of emotional and behavioral support in the group was high, whereby more active children showed a higher level of well-being. Figure 3 presents an illustration. Subsequent analyses showed that the difference between low and high emotional and behavioral support was larger for low active children (estimate = −0.08, *p* = 0.006), whereas for highly active children the difference was not significant (estimate = 0.04, *p* = 0.149).

Finally, the model fit was checked and showed a good model fit for all significant models (CFI = 1.00, TLI = 1.00, RMSEA = 0, SRMR = 0). Table 4 shows the model fit regarding the AIC and BIC, which became better when the moderator variables were included compared to the model fit of model 1, except that the BIC for moderating effect of emotional and behavioral support on the association between activity and well-being became slightly higher.

Alternative models testing moderations of the different temperament scales with staff-child relationship closeness and conflict scale, emotional and behavioral support, and environmental chaos in the group that are not mentioned in Table 4 did not yield significant results (all *p* > 0.05).

## Discussion

Our findings show that children’s temperament is associated with children’s well-being in ECEC and that the level of conflict in the staff-child relationship and a high level of emotional and behavioral support play a role in this association. Regarding the first research question, as to whether there is an association between children’s temperament and well-being in Norwegian ECEC, this study finds that toddlers’ shyness, emotionality, and sociability contribute to their level of well-being in ECEC. Studies on children in ECEC are lacking, but Holder and Klassen’s (2010) results on the relation between temperament and happiness (i.e., subjective well-being and life satisfaction) for children aged 9–12 years are similar to the results of this study. The developers of the EAS Temperament Survey (Buss and Plomin, 1984) suggested that low levels of shyness and high levels of sociability are related to adults’ extraversion and that high levels of emotionality are related to adults’ neuroticism. Extraversion and neuroticism have been found to be strongly related to adults’ happiness by multiple studies (e.g., Furnham and Cheng, 2000; Hills and Argyle, 2001). In addition, activity, or the level of energy, is related to extraversion as well (DeNeve and Cooper, 1998). In this study, the findings for toddlers are to a large extent similar: Children’s shyness and emotionality are negatively related to well-being in ECEC, and sociability is positively related to well-being. However, we did not find a significant association between activity and well-being in ECEC. It might be that less active children also experience well-being in ECEC, as Buss (1981) found that less active children have often more harmonious and peaceful interactions with caregivers.

Our findings are an addition to earlier studies (De Schipper et al., 2003, 2004) that looked at children in ECEC and found that children with a more difficult temperament (e.g., more difficulty to adapt to novelty and showing more irritable distress) showed less well-being in ECEC, whereas children with a more easy-going temperament showed higher levels of well-being. We found that shy and emotional children have more challenges to reach a high level of well-being at the beginning of the childcare year. Extra attention should be paid by the staff to these children’s needs. It might be that some of the more shy and emotional children have difficulty interacting with peers or the staff, as they might be more introverted and susceptible to the atmosphere in the group, especially in novel situations and environments. It could be that these children need support from the staff to feel safe and confident to interact with the others but also need staff to keep track on their need for some time for individual play or for play with fewer peers. Moreover, the staff can help the child to regulate strong emotions and try to find the cause of these emotions. Possibly by discussing the child’s behavior with the parents as well. Children who have an easier temperament by being less shy and emotional and being more social and active seem to have less challenges to reach a high level of well-being. Nevertheless, the staff should also pay attention to more social and active children’s needs. These latter children might cope better with novel situations and environments as they are more open to others, full of energy and/or moving around. This can result in a higher level of well-being at the beginning of the childcare year. Even though social and active children might seem doing fine, they can have periods with lower levels of well-being too. For example, they might need support while playing alone or to regulate their energy levels.

The potential moderating role of the environment brings us to the results pertaining to the second research question, as to whether ECEC process quality moderates the association between children’s temperament and well-being in Norwegian ECEC. Our findings are in line with Thomas and Chess’ (1977) goodness-of-fit theory, which states that children’s outcomes are a result of the interplay between children’s temperament and environment. We find an interplay between children’s temperament and their ECEC environment, affecting children’s well-being in ECEC. Regarding the environment, a distinction can be made between interpersonal relationships and the group. Specifically, this study finds that shy and active children are affected by interpersonal relationships with the staff. Rydell et al. (2005) found that shy children (aged 5 to 6 years) had less close relationships with the staff but also had less conflictual relationships with the staff. However, as this study shows, it might be that shy children experience anxiety at the slightest level of conflict, resulting in less well-being. Active children are typically more extroverted, have a higher energy level, and are searching for play and sensation (DeNeve and Cooper, 1998). This might exceed the caregiver’s toleration threshold for intensity, which sometimes results in more conflictual relationships compared to less active children (Buss, 1981). Nevertheless, even though there was a high level of conflict, more active children showed higher levels of well-being. These findings confirm that shy children are susceptible to the atmosphere and type of interactions with other people. The staff should pay attention when they interact with shy children, as they might feel uncomfortable when interacting with others and possible do not always want to have an interaction. For highly active children, attention should be paid to the quality of the staff-child relationship as well, and conflict should be kept to a minimum.

In addition, low active children seem to profit less from the high emotional and behavioral support in their group, which suggests that they need more individual support to experience well-being in ECEC. This is in line with other studies that showed that high quality in the group does not always support all children, as children have personal needs (Phillips et al., 2012). These findings underline the need for professional caregivers to be responsive and modify the environment to the child’s temperament and needs, and not the other way around (Chess and Thomas, 1984).

Considerable strengths of this study are that we used multiple informant data and multimethod data, which improved the validity. Both the professional caregiver who knew the child best and one of the parents filled out the questionnaires, and the observations were made by external trained and certified observers. In addition, we had a large sample of children, especially for Norwegian standards.

Even though our study has multiple strengths, some limitations need to be mentioned. One limitation is the answer categories for the control variable ‘the child’s number of hours in the ECEC per day’. These answer categories in the questionnaire consisted of both ranges and a specific number of hours. The reason for this is that most children spend at least 6 h per day in Norwegian ECEC centers, so we wanted to have 6 hours as a separate answer category and developed the ranges around these 6 h. However, we did overlook the fact that if a child spends 6 hours per day in ECEC, the parent could have answered the category ‘6 hours’ or ‘6–8 hours’. Therefore, we had to compute these answer categories in our subsequent analyses, which resulted in a few categories with large ranges. This is a limitation, and it would have been better to have more detailed information about the number of hours the child spends in the ECEC center by having more answer categories with smaller ranges. Another limitation of our study is that our sample consisted of ethnic Norwegian children from mainly high-income families. Children from families with a lower income, with a minority language background, and mental/physical disabilities are underrepresented. The latter groups might face more challenges to experience a high level of well-being ECEC, as they might, for example, receive less support from their parents to learn Norwegian, feel excluded, or be restricted due to their disability. In addition, we should note that our sample was represented by children who expressed mainly a high to very high level of well-being. Nevertheless, we do see associations and moderation effects. This underlines the interplay that exists between certain types of temperament and well-being, and the moderation effects of ECEC process quality. At last, the internal consistency of the sociability scale was poor. An explanation might be that some items might apply to both shyness and sociability, as these concepts are intertwined to some extent. Another explanation might be that the items that are focusing on the preference of being or playing alone might not represent “non-sociability.” In Norway, it is common and supported that children explore and do activities on their own, as part of their development.

Future research should follow the children over a longer period as well, so that we can examine if temperament still affects children’s well-being in ECEC over time. It seems more difficult for shy children in particular to experience a high level of well-being at the beginning of the childcare year, but it might be that they express more well-being as they become more familiar with the ECEC setting. In addition, we would be able to examine whether a conflictual staff-child relationship and emotional and behavioral support still moderate the associations or whether a close staff-child relationship or chaos in the group become more important over children’s time in ECEC. Also, potential intervention effects might be investigated to see if the process quality improves in the intervention group compared to the control group, and if these effects moderate the association between children’s temperament and well-being in ECEC.

## Data Availability Statement

The datasets presented in this article are not readily available because the data analyzed in this study is subject to the following licenses/restrictions: We are not allowed to share data outside the key personnel for the grant by the Norwegian Centre for Research Data (NSD). Requests to access the datasets should be directed to EB: elisabet.solheim@r-bup.no.

## Ethics Statement

The studies involving human participants were reviewed and approved by Regional Committees for Medical and Health Research Ethics South East Norway and by the Norwegian Centre for Research Data (NSD), and is registered at clinicaltrials.gov, identifier NCT03879733. Written informed consent to participate in this study was provided by the participants’ legal guardian/next of kin.

## Author Contributions

All authors listed have made a substantial, direct and intellectual contribution to the work, and approved it for publication.

## Conflict of Interest

The authors declare that the research was conducted in the absence of any commercial or financial relationships that could be construed as a potential conflict of interest.

## Publisher’s Note

All claims expressed in this article are solely those of the authors and do not necessarily represent those of their affiliated organizations, or those of the publisher, the editors and the reviewers. Any product that may be evaluated in this article, or claim that may be made by its manufacturer, is not guaranteed or endorsed by the publisher.
